# Heart Failure and Stroke

**DOI:** 10.1007/s11897-018-0405-9

**Published:** 2018-07-30

**Authors:** Katja Schumacher, Jelena Kornej, Eduard Shantsila, Gregory Y. H. Lip

**Affiliations:** 1 Institute of Cardiovascular Sciences, University of Birmingham, Birmingham, UK; 2 Heart Center, Department of Electrophysiology, University of Leipzig, Leipzig, Germany; 3 Institute for Medical Informatics, Statistics and Epidemiology (IMISE), Leipzig University, Leipzig, Germany

**Keywords:** Heart failure, Stroke, Silent atrial fibrillation, Risk stratification, Stroke prevention

## Abstract

**Purpose:**

Ischemic stroke significantly contributes to morbidity and mortality in heart failure (HF). The risk of stroke increases significantly, with coexisting atrial fibrillation (AF). An aggravating factor could be asymptomatic paroxysms of AF (so-called silent AF), and therefore, the risk stratification in these patients remains difficult. This review provides an overview of stroke risk in HF, its risk stratification, and stroke prevention in these patients.

**Recent Findings:**

Stroke risk stratification in HF patients remains an important issue. Recently, the CHA_2_DS_2_-VASc score, originally developed to predict stroke risk in AF patients, had been reported to be a predictive for strokes in HF patients regardless of AF being present. Furthermore, there are several independent risk factors (e.g., hypertension, diabetes mellitus, prior stroke) described.

**Summary:**

Based on the current evidence, HF should be considered as an independent risk factor for stroke. The CHA_2_DS_2_-VASc score might be useful to predict stroke risk in HF patients with or without AF in clinical routine. However, there is only a recommendation for the oral anticoagulation use in patients with concomitant HF and AF, while in patients with HF and no AF, individualized risk stratification is preferred. Current guidelines recommend to prefer non-vitamin Kantagonist anticoagulants over warfarin.

## Introduction

Heart failure (HF) is a frequent condition associated with diverse comorbidities such as cardiac arrhythmias, thromboembolism, impaired renal function, and an increased mortality as a result [1]. The prevalence of HF is approximately 1–2% of the adult population in developed countries with a higher percentage (> 10%) in the population age > 70 years [1].

An increased stroke risk in HF patients has been described in several studies [2]. Pathophysiologically, a predisposition to thromboembolism is caused by abnormal blood flow, abnormal vessel/chamber lining, and abnormal blood particles, also referred to as Virchow’s triad [3]. Abnormal blood flow is evident in patients with HF because of left ventricular systolic dysfunction (LVSD) associated with left ventricular dilatation and abnormal (slowed) blood flow [4]. Given the fact that HF patients with preserved EF (HFpEF) also have an increased stroke risk [5, 6], such patients also exhibit flow abnormalities—apart from vessel wall changes (e.g., endothelial dysfunction) [7, 8] and abnormal blood constituents (e.g., platelet function) [9].

Atrial fibrillation (AF) is the strongest independent risk factor for stroke, followed closely by HF [10]. Of note, HF and AF frequently coexist and exacerbate each other: while AF occurs in more than half (57%) individuals with HF, HF is present in over one third (37%) of AF patients. These results had been shown in 1737 individuals with new AF and 1166 individuals with new HF from Framingham Heart Study [11]. Particularly, paroxysmal AF is mostly associated with stroke in comparison to persistent AF [12]. Problematically, patients are often unaware of these (often asymptomatic) paroxysmal AF attacks and remain underdiagnosed. Indeed, episodes of silent AF are present in approximately one third of the total population of patients with AF [13].

Given the high rates of hospitalization and lethality due to stroke in HF patients, there is a major clinical interest in stroke prediction. Several risk factors associated with an increased stroke risk (e.g., advanced age, prior stroke, diabetes mellitus) [14] have already been identified and were included into different risk models [15•]. The predictive value of the CHA_2_DS_2_-VASc score, originally designed for stroke prediction in AF patients, has also been shown in the HF population [16••, 17].

While oral anticoagulation in AF is recommended dependent on the CHA_2_DS_2_-VASc score, current HF guidelines do not recommend oral anticoagulation for HF patients without documented AF. Indeed, there is an explicit recommendation for an oral anticoagulation only in patients with both HF and AF.

In this review, we discuss the risk of stroke in HF patients, distinguishing between HF with and without coexisting AF. Second, we debate the role of silent AF in these patients and, third, give an overview of risk stratification and therapy approaches.

## Search Strategy

Electronic searches of English literature were performed in the PubMed database for relevant publications from 2000 to 2018 evaluating the risk of stroke in HF patients with and without AF as well as the role of silent AF, possibilities of risk stratification, and therapeutic implications. The following search terms were used in this review: “heart failure” AND/OR “stroke” AND/OR “atrial fibrillation” AND/OR “AF” AND/OR “silent atrial fibrillation” AND/OR “epidemiology” AND/OR “risk stratification” AND/OR “NOAC” AND/OR “warfarin.” Articles were used when studies investigated abovementioned aspects or reviewed the current state of research of stroke in HF. Two authors (K.S. and J.K.) screened all the studies for qualification by abstract screening and full-text reviewing.

## HF Epidemiology

Over 40 million individuals have HF, which is considered as the second most important risk factor for stroke after AF [10, 18]. Of note, 10–24% of patients with stroke have HF, while HF per se (without AF) appears to be the cause of stroke in 9% in comparison to 15% for AF per se and 2% for both HF and AF [19]. As mentioned above, analysis of Framingham Heart Study patients (participants with new-onset AF (*n* = 1737) and/or HF (*n* = 1166)) showed that AF occurs in more than half (57%) of the individuals with HF; HF is presented in over one third (37%) of AF patients [11]. Nevertheless, data reporting the incidence of stroke in HF patients vary among studies with designs and populations [20].

Several clinical trials—*W*arfarin/*A*spirin *S*tudy in *H*eart failure (WASH), *HE*art failure *L*ong-term *A*ntithrombotic *S*tudy (HELAS), *W*arfarin and *A*ntiplatelet *T*herapy in *C*hronic *H*eart failure trial (WATCH), and *W*arfarin versus *A*spirin in *R*educed *C*ardiac *E*jection *F*raction trial (WARCEF)—investigating HF patients in sinus rhythm have reported a low incidence of stroke in their populations [21–23]. In the WATCH trial, the incidence of stroke ranged from 0.4% in the warfarin group to 2.3% in the aspirin plus clopidogrel group. In a community-based cohort of 630 patients, Witt et al. found that 16% of the HF patients (where 41% had AF) experienced an ischemic stroke [2]. Their stroke risk was 17.4-fold increased within first 30 days after the initial diagnosis and remained elevated during follow-up of 5 years [2]. In another study, Mujib reported an approximately 1% annual rate of stroke in HF patients with sinus rhythm, which was higher than in general population (0.3%) [24] but lower than in those with both HF and AF. The presence of HF is associated with high mortality and hospitalization rates. Indeed, stroke patients with HF have longer hospitalization periods and a 2.0–2.5-fold higher mortality than patients without HF [2]. Stroke risk in HF patients seems to depend on HF severity: mild to moderate HF is associated with an annual stroke risk of 1.5% [25, 26], while stroke risk in severe HF approaches 4% [27].

As mentioned, concomitant HF and AF are the cause of 2% of all strokes. The overall rate of stroke in HF without AF (1.6% per year) is about one third of that seen in AF without HF (5%) [19]. Of note, AF type could play an important role for the stroke occurrence in HF patients. However, the literature is controversial. On the one hand, persistent AF is described to not increase stroke risk in contrast to paroxysmal AF [12]. On the other hand, several studies reported an equal risk of stroke for paroxysmal and persistent AF [28] or even opposite results [29]. A meta-analysis including 18 papers with 134,847 AF patients [30] showed that the stroke risk was higher in patients with persistent AF with ORs of 0.75 (95% confidence interval (CI) 0.61–0.93) in studies with no oral anticoagulants and 0.77 (95% CI 0.68–0.88) in studies with oral anticoagulants in all patients. Nevertheless, it remains unclear if AF type is an independent predictor of stroke or predicated on a different patient profile regarding risk factors and comorbidities [31]. Patients with paroxysmal AF are likely to be younger, with a lower prevalence of structural heart disease, major comorbidities, and also have lower estimated thromboembolic and bleeding risks [32]. Based on this knowledge, it seems more reasonable that persistent AF has the higher stroke risk. But paroxysmal AF remains often asymptomatic as well as undiagnosed and consequently untreated leading to a possible increased risk of cardioembolic events [33].

Four randomized clinical trials investigating the effect of non-vitamin K antagonist oral anticoagulants (NOACs) anticoagulants (NOACs) in AF patients have presented different data on the effect of concomitant HF and AF. Whereas the Apixaban for Reduction in Stroke and Other Thromboembolic Events in Atrial Fibrillation (ARISTOTLE) study [34] and the Rivaroxaban versus Warfarin in Nonvalvular Atrial Fibrillation (ROCKET AF) study [35] could not find a significant difference in risk rates for stroke in AF patients with and without HF, the Effective aNticoaGulation with factor xA next GEneration in Atrial Fibrillation–Thrombolysis In Myocardial Infarction study 48 (ENGAGE AF TIMI 48) trial found an increased risk for patients with both AF and HF present [36]. In the Randomized Evaluation of Long-Term Anticoagulation Therapy (RE-LY) trial, there was a numerically higher incidence of stroke in patients with AF and HF compared to AF without HF, but this was non-significant after multivariable adjustment [37].

While both HF and AF are independent risk factors for stroke, the coexistence of both diseases increased the risk even more. Kang et al. reported a 3.5-fold increased risk for stroke in HF-only patients, while patients with HF + AF had a fivefold risk in stroke [38]. A more recent study did not find any significant difference in stroke risk between HF patients with or without AF (incidence = 2.6% patients with AF vs 2.8% without AF) [39]. The presence of AF had been also attributed to play a role in stroke etiology, as patients with both HF and AF mostly experienced cardioembolic strokes regardless of the HF etiology. Of note, patients with HF but without AF have different stroke causes according to the HF etiology: for example, patients with dilated cardiomyopathy or valvular heart disease had more frequent cardioembolic strokes while those with coronary artery disease/hypertension tended to experience atherosclerotic and lacunar strokes [40].

## Heart Failure with Preserved Ejection Fraction

Most of prior studies investigated the stroke risk in patients with HF and reduced ejection fraction (HFrEF); however, HF with preserved EF (HFpEF) had an increased risk for strokes as well [5, 6]. Studies investigating the stroke risk in patients with HFpEF in comparison to HFrEF have generally found a similar stroke risk [41–45]. In contrast to HFpEF, the patients with HFrEF have a higher mortality [44, 45]. Cogswell et al. hypothesized a possible influence of undiagnosed (silent) paroxysmal AF on stroke risk in HFpEF patients, given that stroke risk in patients with HFpEF without AF and HFpEF with AF as well as AF-only was similar [5].

## Silent Atrial fibrillation in HF

Atrial fibrillation is the most common cardiac arrhythmia [46] and the strongest risk factor for the thromboembolic stroke [10]. Because of a high prevalence of paroxysmal AF in patients with acute stroke [12], more extensive diagnostic approaches to reveal paroxysmal AF episodes are needed [47]. This is aggravated by the fact that one third of patients with AF are not aware of its presence; hence, the term “silent AF” has been introduced.

Silent AF is often discovered after serious cerebro- and cardiovascular complications such as ischemic stroke and HF via routine self-monitoring of the pulse, 12-lead electrocardiogram (ECG), 24-h Holter ECG [13], implanted pacemakers, and defibrillators. In this context, attention has been directed towards AF burden, defined by time spent in AF per unit of time [48]. Several studies analyzing implanted devices showed that 20–42% of HF patients have silent AF episodes [49–51]. Silent AF was also common (10%) at the acute phase of ischemic stroke or transient ischemic attacks (TIAs) [52]; 46% of patients suffering a cryptogenic stroke had silent AF on continuous electrocardiographic monitoring [33]. Of note, stroke incidence in silent AF is significantly higher in patients with multiple risk factors, especially hypertension, advanced age, obesity, diabetes mellitus, smoking, and previous cardiac disease [53–55] and in those with higher CHA_2_DS_2_-VASc score [56].

The presence of silent AF had been also described in patients with coronary artery disease and myocardial infarction [57]. Turakhia et al. found a threefold higher rate of cardiovascular death and a fivefold higher rate of hospitalization for HF in patients with silent AF [58]. In this context, silent AF was also common after coronary artery bypass grafting (a third had recorded AF episodes) [59]. The fact that silent AF is a common finding in different populations leads to the assumption that it could also play a role in stroke development in HF patients.

## Risk Stratification of Stroke in HF

**Fig. 1:**
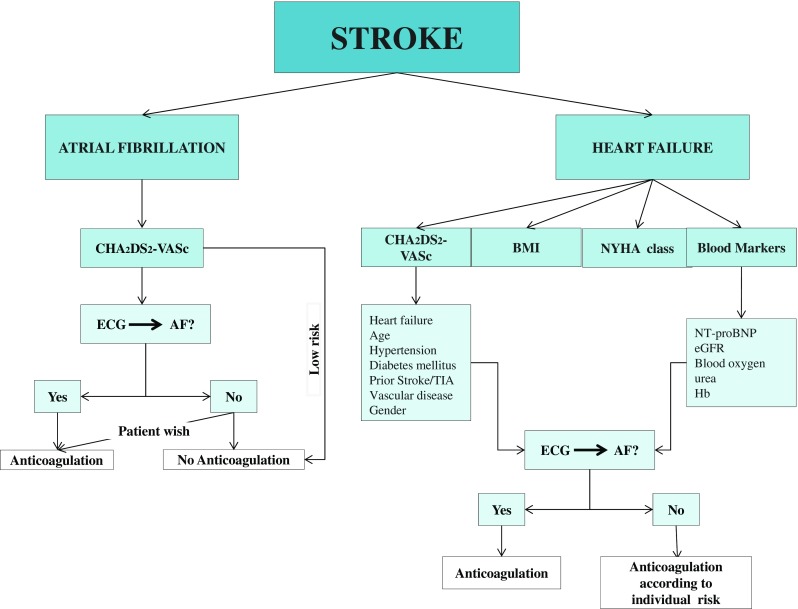
Risk factors for stroke in patients with heart failure. Abbreviations: AF, atrial fibrillation; BMI, body mass index; NYHA class, New York Heart Association class; TIA, transient ischemic attack; NT-proBNP, N-terminal prohormone of brain natriuretic peptide; eGFR, estimated glomerular filtration rate; Hb, hemoglobin

Because of the high prevalence of HF in the population and the associated stroke risk, there is interest in stroke prediction and evaluation of the possible need of antithrombotic therapy (Fig. 1).

The CHA_2_DS_2_-VASc score is widely used to estimate the risk of stroke in AF patients and to help in decision-making regarding oral anticoagulation [60]. In a nationwide prospective cohort of 42.987 patients with HF, Melgaard et al. demonstrated that CHA_2_DS_2_-VASc score has also predictive power for stroke, regardless of AF presence [16••]. Similar results have been found by Wolsk and colleagues in the Danish registry of 136,545 HF patients (with or without AF) [17] and in the WARCEF cohort [61]. The studies support consideration of the CHA_2_DS_2_-VASc score for prediction of the risk of stroke in HF irrespective of AF presence. Indeed, several studies examined the components of the CHA_2_DS_2_-VASc score and demonstrated their individual association with stroke in HF: congestive HF represented by a decreased ejection fraction (hazard ratio [HR] 0.98–2.15) [15•, 34, 62, 63], hypertension (HR 1.18) [15•, 62, 64, 65], age (HR 1.34–1.35) [14, 15•, 62–64], diabetes mellitus (HR 1.114–1.87) [14, 15•, 16••, 62, 63, 65], prior stroke/TIA (HR 1.81–2.68) [14, 15•, 39, 63, 64], vascular disease (HR 1.34) [66], and gender (HR 0.569) [15•, 62, 63]. In WARCEF sub-study with patients with sinus rhythm, the ejection fraction was associated with stroke only if its baseline values were less than 15% [62, 63].

However, the data are inconsistent. For example, McMurray et al. did not find a correlation between ejection fraction and stroke risk despite numerically higher rate of stroke and systemic embolism in patients with left ventricular systolic dysfunction [34]. Prior stroke [14, 15•, 39, 62, 64], gender [15•], and also peripheral artery disease [66] are associated with stroke risk in HF patients. Nevertheless, the correlation between stroke risk and age [14, 15•, 64] in HF patients as well as those with diabetes mellitus [15•, 16••, 65] and hypertension are conflicting [62].

Although there are many different scores predicting the mortality in HF [67–69], the CHA_2_DS_2_-VASc score is the only one shown to be useful for stroke prediction in HF. Due to the lack of a convenient and accurate model to predict stroke and the accompanied increased mortality in HF, Freudenberger et al. proposed a new scoring system for stroke prediction in patients with an ejection fraction of less 35%, with a full model of their score, including 14 risk factors, and to provide better clinical practicability a simpler more practical score of only eight of these components: age, blood oxygen urea, ejection fraction, hemoglobin, gender, diastolic blood pressure, diabetes mellitus, and prior stroke. In their study population (*n* = 2305), the new developed score performed modestly but was superior (statistically) to CHA_2_DS_2_-VASc score in stroke prediction (area under the curve [AUC] 0.660, 95% CI 0.58–0.74 vs 0.52, 95% CI 0.398–0.63, *p* = 0.001) [15•].

Several studies investigated the impact of renal function on stroke risk in HF. Melgaard et al. showed an increased risk of ischemic stroke and intracranial bleeding in HF patients with stable chronic kidney disease, but this association could only be found in patients without renal replacement therapy [70]. These findings are in agreement with the results of another study showing an association between estimated glomerular filtration rate and stroke risk in HF patients [64].

## Therapy

Given the increased risk of thromboembolic complications in patients with HF, anticoagulation should be considered in these patients also in the absence of AF. Nevertheless, current guidelines do not recommend anticoagulation for patients with HF in general [1].

### Vitamin K Antagonists

There are four randomized clinical trials investigating the effect of Warfarin on stroke risk in patients with HF in comparison to aspirin: WASH [21], HELAS [22], WATCH [23], and WARCEF [71]. Details of the trials are summarized in Table 1.

**Table 1:** Warfarin vs antiplatelet therapy in patients with sinus rhythm

	WASH	HELAS	WATCH	WARCEF
Year of publication	2004	2006	2009	2012
Number of patients	279	197	1587	2305
Treatment arms	Aspirin vs warfarin Placebo 99 Aspirin (300 mg) 91 Warfarin (INR 2–3) 89	Aspirin vs warfarin Ischemic Heart disease: 61 Aspirin (325 mg) 54 Warfarin (INR 2–3) Dilatative cardiomyopathy: 38 Warfarin (INR 2–3) 44 Placebo	Aspirin/clopidogrel vs warfarin 523 Aspirin (162 mg) 524 Clopidogrel (75 mg) 540 Warfarin (INR 2.5–3)	Aspirin vs warfarin 1163 Aspirin (325 mg) 1142 Warfarin (INR 2.5–3)
AF	ca. 6% (baseline)	None (exclusion criteria, patients with AF in follow-up were withdrawn)	10% (follow-up)	ca. 4% (baseline)
Follow-up (mean)	27 months	ca. 20 months	21 months	3.5 years
Primary endpoints	Composite of (1) death (2) non-fatal myocardial infarction (3) non-fatal stroke	Composite of (1) non-fatal stroke (2) peripheral or pulmonary embolism (3) myocardial (re)infarction (4) re-hospitalization (5) exacerbation of heart failure (6) death from any cause	Composite of (1) all-cause mortality (2) non-fatal myocardial infarction (3) non-fatal stroke	Composite of (1) ischemic stroke (2) intracerebral hemorrhage (3) death from any cause
Secondary endpoints	(1) Death or cardiovascular hospitalization (incl. major hemorrhage) (2) Death or all-cause hospitalization (3) Total number of hospitalization (4) Composite of death, cardiovascular hospitalization and increase in diuretic therapy for worsening heart failure	(1) Cardiac and total mortality (2) Myocardial infarction or re-infarction (3) Heart failure exacerbation	(1) All-cause mortality (2) Nonfatal myocardial infarction (3) Nonfatal stroke (4) Hospitalization for heart failure	Composite of (1) primary outcome (2) myocardial infarction (3) hospitalization for heart failure
Safety endpoints	Included in secondary endpoints	Intracranial hemorrhage, incidence of bleeding while on study drug, differences in bleeding index on study drug	Major bleeding	Major bleeding, minor bleeding
Results	Neither warfarin nor aspirin reduces risk of stroke in patients with HF	Neither warfarin nor aspirin reduced risk of stroke in patients with HF and without AF	Warfarin reduced stroke more than aspirin or clopidogrel but with a higher risk of bleeding	Warfarin was superior to aspirin concerning ischemic stroke but is accompanied with higher rates of intracerebral hemorrhages

The WASH and HELAS trials were small studies, which were underpowered but showed no suggestion for the efficacy of anticoagulant therapy for HF patients in sinus rhythm [21, 22, 72]. The WATCH and WARCEF trials were larger studies (with WARCEF being a double-blind trial) and showed no significant benefit for the primary outcome that included mortality but a significant risk reduction for stroke (a secondary outcome) in patients treated with warfarin compared to aspirin; however, the positive effect was neutralized by an increased risk of major bleeding [23, 71]. In WATCH, clopidogrel was superior neither to warfarin nor to aspirin [23].

A meta-analysis of these four trials based on 3665 patients showed that warfarin reduced the risk of cardiovascular events including stroke by 20% compared to antiplatelet therapy (risk ratio (RR) 0.79, 95% CI 0.63–1.00; *I*^2^ = 0%), but the risk of major bleeding was twofold higher (RR 2.00, 95% CI 1.44–2.78; *I*^2^ = 4%). Consequently, the stroke risk reduction of warfarin was outweighed by the increased bleeding risk [73••]. Interestingly, there was no significant increase of intracranial hemorrhage on warfarin compared to antiplatelet therapy [74].

### Non-Vitamin K Antagonists

The efficacy and safety of these anticoagulation drugs were shown in AF patients in four randomized double-blind trials: RE-LY, ARISTOTLE, ROCKET AF, and ENGAGE AF [75–78].

In subgroup analyses, the effect of NOACs had been investigated in AF patients with and without HF (Table 2). In summary, NOACs (dabigatran [37], apixaban [34], or at least non-inferior rivaroxaban [35] and edoxaban [36]) showed relative efficacy and safety compared to warfarin; however, there were no differences between patients with and without HF. Based on these results, current HF management guidelines recommend to prefer NOACs over warfarin in patients with concomitant HF and AF [1].

**Table 2:** Efficacy and safety of non-vitamin K antagonist oral anticoagulants in patients with atrial fibrillation and heart failure

Sub-studies	RE-LY	ARISTOTLE	ROCKET-AF	ENGAGE AF
Year of publication	2013	2013	2013	2016
Number of patients	18.113 4.904 with HF 13.209 without HF	14.671 3.207 with HF (EF > 40%) 2736 with HF (EF < 40%) 8728 without HF	14.171 9.033 with HF 5.138 without HF	14.071 6.344 HF HYHA I–II 1801 NYHA III–IV 5.926 without HF
Treatment arms	Dabigatran vs warfarin	Apixaban vs warfarin	Rivaroxaban vs warfarin	Edoxaban vs warfarin
Follow-up (median)	2.0 years	18 months	707 days	2.8 years
Primary endpoints	(1) Stroke (ischemic or hemorrhagic) (2) Systemic embolism	(1) Stroke (ischemic or hemorrhagic) (2) Systemic embolism	(1) Stroke (ischemic or hemorrhagic) (2) Noncentral nervous system embolism	(1) Stroke (ischemic or hemorrhagic) (2) Systemic embolism
Secondary endpoints	(1) Vascular death (2) Hospitalization (3) Intracranial bleeding (4) Total bleeding	(1) Composite of - Stroke - Systemic embolism - Death (2) Net clinical benefit composite of - Stroke - Systemic embolism - Major bleeding - Death from any cause	(1) All-cause death (2) Myocardial infarction (3) Composite of - Stroke - Systemic embolism - Vascular death	(1) Ischemic stroke (2) Hemorrhagic stroke (3) Cardiovascular death (4) Cardiovascular hospitalization (5) All-cause death
Safety endpoints	Major bleeding	Major bleeding	(1) Primary: major or non-major clinical relevant bleeding (2) Secondary: intracranial hemorrhage and hemorrhagic stroke	Major bleeding
Results	Dabigatran was superior to warfarin concerning stroke (annual rate 1.44 vs 1.92%) and bleeding risk (annual rate 3.10 vs 3.90%). No differences in efficacy and safety between HF and No-HF	Apixaban reduced risk for stroke (HR 0.89, 95% CI 0.81–0.98)/bleeding/death (HR 0.85, 95% CI 0.78–0.92) more than warfarin independently of presence of HF	Rivaroxaban was non-inferior to warfarin concerning efficacy (HR 0.94, 95% CI 0.76–1.17) and safety (HR 1.05, 95% CI 0.95–1.15) there was no difference between HF and No-HF	Edoxaban was non-inferior to warfarin concerning efficacy (stroke in no HF: HR 0.87, 95% CI 0.69–1.11, NYHA III–IV: HR 0.83, 95% CI 0.55–1.25) and even more safe (major bleeding in no-HF: HR 0.82, 95% CI 0.68–0.99, NYHA III–IV: HR 0.79, 95% CI 0.54–1.17), there was no difference between HF and No-HF

A meta-analysis of RE-LY, ARISTOTLE, and ROCKET AF including 19,122 subjects showed a significant risk reduction for stroke in patients with both HF and AF combined with a decreased bleeding risk; in HF patients, NOACs were similar effective or even safer compared to those without HF [79].

However, it remains unclear whether NOACs have a positive impact of stroke risk reduction in patients with HF but in sinus rhythm. This question had been addressed in a randomized, double-blind, placebo-controlled trial (COMMANDER HF) investigating the efficacy and safety of rivaroxaban vs placebo in HF patients without AF, where HF is related to ischemic heart disease and all patients are taking aspirin therapy [80].

## Current Approach

Based on RE-LY, ARISTOTLE, ROCKET AF, and ENGAGE AF, European HF management guidelines recommend anticoagulation in patients with both HF and AF, with a preference for NOACs [1]. Because of an increased bleeding risk outweighing the stroke risk reduction using warfarin in patients with HF but without AF [20–23, 71], the therapy of these patients needs to be tailored to the individual risk profile (e.g., prior stroke, cardiac thrombi) [1].

## Conclusions

Based on the current evidence, HF should be considered as an independent risk factor for stroke. The CHA_2_DS_2_-VASc score might be useful to predict stroke risk in HF patients with or without AF in clinical routine.

Thus far, there is only a recommendation for the oral anticoagulation use in patients with concomitant HF and AF, while in patients with HF and no AF, individualized risk stratification is preferred. Based on recent data, NOACs should be preferred over warfarin. Finally, the results of ongoing studies may clarify further aspects of anticoagulation in HF patients without AF.
